# Time to Recovery From Moderate Acute Malnutrition and Its Predictors Among Children 6–59 Months of Age Enrolled in Targeted Supplementary Feeding Program in Darolebu District, Eastern Ethiopia: A Retrospective Cohort Study

**DOI:** 10.3389/fpubh.2022.914837

**Published:** 2022-07-14

**Authors:** Mohammed Yahya Rashid, Jemal Yusuf Kebira, Lemessa Oljira, Merga Dheresa

**Affiliations:** ^1^Child Survival Initiatives, Harar, Ethiopia; ^2^School of Public Health, College of Health and Medical Sciences, Haramaya University, Harar, Ethiopia; ^3^School Nursing and Midwifery, College of Health and Medical Sciences, Haramaya University, Harar, Ethiopia

**Keywords:** moderate acute malnutrition, treatment outcome, time to recovery, predictors, Eastern Ethiopia

## Abstract

**Background:**

Acute malnutrition is a major global public health problem, particularly in low and middle-income countries. A targeted supplementary feeding program is an approach recommended to address moderate acute malnutrition in food insecure settings. Preventing and treating moderate acute malnutrition requires identifying factors shown to affect the treatment outcome and duration of stay on treatment. This study aimed to determine the treatment outcome and predictors of recovery time from moderate acute malnutrition among children 6–59 months of age in Darolebu district, Eastern Ethiopia.

**Methods:**

A retrospective cohort study design was conducted on 540 children with moderate acute malnutrition. A Kaplan–Meier survival analysis was used to estimate the recovery time. Cox proportional hazard regression model was used to determine the association between the independent and the outcome variables. The proportional hazard assumption of the model was checked graphically and statistically. Any violation of the proportional hazard assumption of the model was also considered and adjusted in the analysis. Finally, a variable with a *P-*value <0.05 in the multivariate cox regression model was considered statistically significant.

**Results:**

The overall recovery rate was 73% (95% CI 69.4–76.4%) with the median time to recovery of 16 weeks. Being between the ages of 24 and 59 months (AHR = 1.24, 95% CI: 1.01–1.54), having a mid-upper arm circumference (MUAC) at admission between 11.5 and 11.9 cm (AHR = 1.27, 95% CI: 1.34–2.61), walking for an hour or less to receive services (AHR = 1.2, 95% CI: 1.02–1.89), using ready-to-use supplementary food (AHR= 1.8, 95%CI: 1.38–2.39) were significant predictors of recovery time.

**Conclusion:**

The recovery rate was slightly below the accepted minimum international standard, suggesting that further work is needed to improve the treatment outcomes and mortality and morbidity associated with moderate acute malnutrition.

## Introduction

Moderate acute malnutrition (MAM), defined as having a weight-for-height *Z-*score (WHZ) −3 and −2, without edema and/or mid-upper arm circumference (MUAC) between ≥11.5 and <12.5 cm (1), is associated with increased mortality and morbidity among children under 5 years of age (2, 3). Children with MAM face a greater risk of morbidity from infectious diseases, weakened immune systems, and impaired physical and cognitive developments (3–5). Moreover, beyond deterioration to severe acute malnutrition, a recent study suggested that repeated episodes of moderate acute malnutrition in children can eventually lead to stunting over time with irreversible effects and affect intergenerational growth failure (6).

Globally, an estimated 47 million children younger than 5 years had acute malnutrition, of which 70% were attributed to moderate acute malnutrition (7). Africa and Asia bear the greatest share of moderate acute malnutrition (3). In 2019, Asia and Africa account for more than two-thirds (69 and 27%, respectively) of all wasted children under the age of five. Consequently, Southern Asia is the sub-region with the highest prevalence of moderate wasting (14.3%) followed by Oceania (9.5%), in the world (7).

Ethiopia has made substantial progress in reducing the prevalence of malnutrition in the past two decades (8, 9). However, childhood undernutrition, particularly moderate acute malnutrition, remains a major challenge (10, 11) as the latest national survey indicated, 10% and 7% of children under five years of age were moderately malnourished in 2016 and 2019, respectively (11, 12). Moreover, the progress made has been uneven across the administrative regions of Ethiopia as the prevalence of moderate acute malnutrition ranges from the highest of 21% in Somalia to the lowest of 2% in Addis Ababa (11).

Currently, the most common intervention for the management of MAM is the targeted supplementary feeding program (TSFP) (13). The targeted supplementary feeding program is a management approach intended to address the immediate and long-term impacts of MAM in food insecure settings (14). The program linked community-and facility-based approaches and was characterized by nutritional monitoring, active case finding, distributions of nutritious food supplements, and routine medical treatment for vulnerable children (15–17).

As a part of the strategy to meet the sustainable development goal target of reducing child mortality and morbidity attributed to malnutrition, the government of Ethiopia launched a high-level collaborative platform, the “Seqota declaration” which aimed to end child undernutrition by 2030 (18). Further to this declaration, the country launched the second national nutrition program and a new health sector transformation plan (HSTP) that constitute the settings for the management of moderate acute malnutrition through targeted supplementary feeding programs (19, 20).

Preventing and treating MAM requires understanding the factors shown to affect the management outcomes and duration of stay on treatment (21, 22). Previous studies conducted in various regions of Ethiopia have been focused on the management of severe acute malnutrition (23–27). However, the management of moderate acute malnutrition should also be public health and development priority (28, 29). Indeed, generating evidence on more effective programmatic approaches to manage MAM is imperative for the continued efforts to tackle child undernutrition in Ethiopia. Therefore, this study aimed to determine the treatment outcome and predictors of recovery time from moderate acute malnutrition among children 6–59 months of age enrolled in TSFP in Darolebu district, Eastern Ethiopia.

## Methods and Materials

### Study Design, Setting, and Period

Institutional-based retrospective cohort study design was conducted on MAM children who were enrolled in a targeted supplementary feeding program in Darolabu district, Eastern Ethiopia from July to December 2020. Data were collected from January 15 to 30, 2021. The district is situated in the western Harare zone, eastern Ethiopia, and is classified as a food-insecure district; thus eligible for a targeted supplementary feeding program. The total population (2019 projection based on the 2007 Census, CSA) of the district was 192, 922. For administrative purposes, the district is subdivided into 41 kebeles (the smallest administrative unit in Ethiopia), 37 rural, and 4 urban kebeles. Regarding the health facilities, the district has 41 health posts, 6 health centers, and 1 primary hospital.

### Inclusion and Exclusion Criteria

A total of 540 children who met the eligibility criteria were included in the study. Children aged 6–59 months, with admission MUACs of 11.0 and 12.0 cm, and a full record report were included in the study, whereas children whose date of birth was missed by the primary caregivers and those with an incomplete record were excluded.

### Sample Size Determination and Sampling Technique

Taking some variables into account from previous similar studies, the sample size for this study was determined using EPI-Info version 7 statistical software, considering the following assumptions: 95%CI, 80% power of the study, exposed to non-exposed ratio of 1:1, and a 5% incomplete record/non-response rate. Accordingly, seven variables were considered, and we selected the “food sharing” variable since it yielded the largest sample size as depicted in Table 1. Finally, we considered a 567 sample size for the study. Then, a multi-stage sampling technique was used to select the study participants. There are 41 health posts in the district. The populations living around these health posts were assumed to be homogenous and to be taking benefits of the supplementary feeding program. In addition, the supplementary food program protocol for the management of MAM works equally well at all health post levels. So, initially, eight health posts were selected randomly. Next, a sampling frame of children 6–59 months of age managed for MAM under a targeted supplementary feeding program in each health post was prepared by reviewing the registration book. Then, total samples were distributed proportionally to each health post using the probability proportional sampling technique. Finally, the children were selected by systematic random sampling from each health post based on their unique registration number.

**Table 1:** Sample size estimation for time to recovery from moderate acute malnutrition and its predictors among children 6–59 months of age enrolled in targeted supplementary feeding program in Darolebu district, eastern Ethiopia, 2020.

**Variables**	**Proportion of exposed**	**Proportion of non exposed**	**Total sample size**	**Reference**
Food sharing	43.8	56.2	567	(30)
Vitamin A supplement	60.6	48	546	(10)
Deworming	89.1	72.1	202	(31)
Overcrowding	70	30	61	(30)
Parental alcohol use	83	72.6	565	(31)
Family food insecurity	49.3	62.9	468	(10)
Access to transportation	58.5	41.5	209	(30)

### Data Collection Tools and Procedures

Data were collected by record review and a face-to-face interviewer-administered questionnaire. Data about the characteristics of the children were extracted from the registration book. On the other hand, the questionnaire used to collect data from the primary caregiver was designed by reviewing related literature (30–32) and adapted to the local situation. Eight health extension workers and three nursing professionals, a total of eleven personnel, have participated in the data collection and supervision processes, respectively. Children's household addresses were traced in the children's parent database available at each health post. Following recruitment, information has been provided about the data collection procedure and re-visit for closed houses on the second day and considering a replacement if they are absent on the second day. The interview consisted of the study subjects' socio-demographic characteristics, a household-related condition, routine medication, supplements, therapeutic feeding, and co-morbidity-related condition.

### Data Quality Assurance

Data quality assurance mechanisms were carefully developed and implemented at various stages of the study. Initially, the questionnaire was prepared in English language and then translated into the local language (Afan Oromo). Then, it was pretested on 5% of the sample size in the district's non-studied health post (Buraysa Health Post) 2 weeks before actual data collection commencement. After the pre-test, some modifications were made to the initially prepared questionnaire. Data collectors and supervisors were trained for 2 days on the purpose of the study, data collection procedures, and supervision activities. The collected data were checked for completeness and consistency daily. Double data entry was done by two data clerks and cross-checked to ensure consistency. The principal investigator and supervisors coordinated all data collection processes and have made immediate corrections accordingly.

### Operational Definitions

*Recovered*: a child who left the program with anthropometric improvement (reaching the target weight of 13% of admission weight and MUAC ≥11.9 cm or > −2 *Z-*score).

*Defaulter*: a child absent from the program for three consecutive distributions.

*Non-responder*: a child who could not meet discharge criteria over 4 months.

*Recovery time*: the time from admission to the program until the child is discharged after being claimed to have recovered from MAM.

*Re-admission*: a child recovered from MAM and was again admitted to the program within 3 months.

### Data Processing and Analysis

Collected data were coded, cleaned, and entered using Epi Data version 3.1 software package and exported into Statistical Package for Social Science (SPSS) version 23 for analysis. Data cleaning was performed to identify outliers/inconsistencies, errors, and missing values. Both descriptive and analytical statistics were executed. Cross tabulation, frequency, and percentage for categorical variables were used to report the descriptive data. Mean (SD), and median (IQR) for continuous variables were also used to describe the study subjects. The primary outcome variable was time to recovery. A Kaplan–Meier survival analysis was used to estimate the recovery time. Cox proportional hazard regression model was used to determine the association between the independent and the outcome variables. First, a bivariate Cox proportional hazard regression analysis was performed. Next, all covariates that had an association with the outcome variable at a *p*-value of 0.05 or less were retained and entered into the multivariable cox regression model. The crude hazard ratio (CHR) and adjusted hazard ratio (AHR) were estimated with 95% confidence intervals (CI) for bivariate and multivariate, respectively. The proportional hazard assumption of the model was checked using a graphical evaluation of the Kaplan–Meier curve and Schoenfeld residuals. Any violation of the proportional hazard assumption of the model was also considered and adjusted in the analysis. Finally, a *P*-value <0.05 was used to declare the presence of a significant association between independent and outcome variables.

## Results

### Socio-Demographic Characteristics of the Study Participants

A total of 540 children 6–59 months of age were included in the study and yielding a 95% response rate. The mean age of children at admission was 18.2 months. Males accounted for more than half, 280(51.9%) and 282(52.2%) of the children were <2 years of age. Three-quarters (75%), of the study subjects, were rural residents. Most of the study subjects, 501(92.8%) and 527(97.6%), belonged to Oromo ethnic groups and the Muslim religion, respectively. Four hundred and eighty-four (89.6%) study subjects were new admissions to the treatment program, and 341(63.1%) weighed ≥7 Kg at admission. The majority, 478(88.5%), of children's primary caregivers were housewives (Table 2).

**Table 2:** Socio-demographic characteristics of children 6–59 months of age with moderate acute malnutrition enrolled in targeted supplementary feeding program in Darolebu district, Oromia region, Eastern Ethiopia, 2020 (*N* = 540).

**Variables**	**Frequency**	**Percent**
**Age (in months)**		
6–23	282	52.2
24–29	258	47.8
**Sex of the child**		
Male	280	51.9
Female	260	48.1
**Residence**		
Urban	135	25.0
Rural	405	75.0
**Ethnicity**		
Oromo	501	92.8
Others	39	7.2
**The religion of the primary caregiver**		
Muslim	527	97.6
Non-Muslim	13	2.4
**Educational status of the caregiver**		
No formal education	281	52.0
Primary school	168	31.1
Secondary school and above	91	16.9
**Occupation of caregiver**		
Housewife	478	88.5
Civil servant	9	1.7
Merchant	53	9.8
**Responsible person with primary child care**		
Mother	427	79.1
Father	113	20.9
**Household member**		
1–5 members	166	30.7
≥6 members	374	69.3
**Admission MUAC (in centimeter)**		
11–11.4 cm	300	55.5
≥11.5 cm	270	44.5
**Admission status**		
New admission	484	89.6
Re-admission	56	10.4
**Weight at admission (in kilogram)**		
<7kg	199	36.9
≥7kg	341	63.1

Three hundred and seventy-four (69.3%) study subjects live with six or more family members. Based on the household food insecurity access scale assessment, over two-thirds, 371(68.7%), of households were categorized as having food insecurity. Concerning food assistance, 145(26.9%) households were beneficiaries of food from another program. More than half, 293(54.3%) and 231(42.8%), of households, have a latrine and use tap water, respectively. Nearly half (46.7%) of the houses had two rooms. More than a third (37%) of the households reported sharing nutritious foods with other family members. Nearly a third (34.6%) of respondents had walked for more than an hour to receive the services from the nearby health post (Table 3).

**Table 3:** Household characteristics of children 6–59 months of age with moderate acute malnutrition enrolled in targeted supplementary feeding program in Darolebu district, Oromia Region, Eastern Ethiopia, 2020 (*N* = 540).

**Characteristics**	**Frequency**	**Percent**
**Household member**		
1–5 members	166	30.7
≥6 members	374	69.3
**Household food security status**		
Food secured	169	31.3
Food insecure	371	68.7
**Other programs provide food assistance to the household**		
Yes	145	26.9
No	395	73.1
**Latrine availability**		
Yes	293	54.3
No	247	45.7
**Source of water**		
Tap water	231	42.8
Protected spring	101	18.7
Unprotected spring	94	17.4
Pond water	114	21.1
**Shared nutritious food with other family members**		
Yes	200	37
No	340	63
**Time taken to receive services from the nearby health post**		
≤ 60 min	353	64.5
>60 min	187	34.6
**Number of rooms in the house**		
One	164	30.3
Two	252	46.7
Three and above	124	23

### Child's Health Services

More than two-thirds, 380(70.4%), of the study subjects took vitamin-A supplements, and 343(63.5%), took de-worming medication. Most of the children, 396 (83.3%), used ready-to-use supplementary food (RUSF). Three hundred and forty-five, (63.9%) children had received the measles vaccination. Almost, three-quarters (74.1%), and 77.4% of the study subjects had practiced exclusive and complementary breastfeeding, respectively. Diarrhea was the most prevalent (39.6%) reported co-morbidity, followed by cough (16.1%) among the study subjects. Fever was the least (11.1%) co-morbidity reported among the study participants (Table 4).

**Table 4:** Health services characteristics of children 6–59 months of age enrolled in targeted supplementary feeding program in Darolebu district, Oromia Region, Eastern Ethiopia, 2020 (*N* = 540).

**Variables**	**Frequency**	**Percent**
**Received Vitamin-A supplements**		
Yes	380	70.4
No	160	29.6
**Deworming**		
Yes	343	63.5
No	197	36.5
**Received Measles vaccination**		
Yes	345	63.9
No	195	36.1
**Exclusive breastfeeding**		
Yes	400	74.1
No	140	25.9
**Complementary feeding**		
Yes	418	77.4
No	122	22.6
**Specialized nutritious foods used**		
CSB++	144	26.7
RUSF	396	73.3
**Diarrhea**		
Yes	214	39.6
No	326	60.4
**Fever**		
Yes	60	11.1
No	480	88.9
**Cough**		
Yes	87	16.1
No	453	83.9

### Treatment Outcome and Median Time to Recovery From Moderate Acute Malnutrition

**Figure 1:**
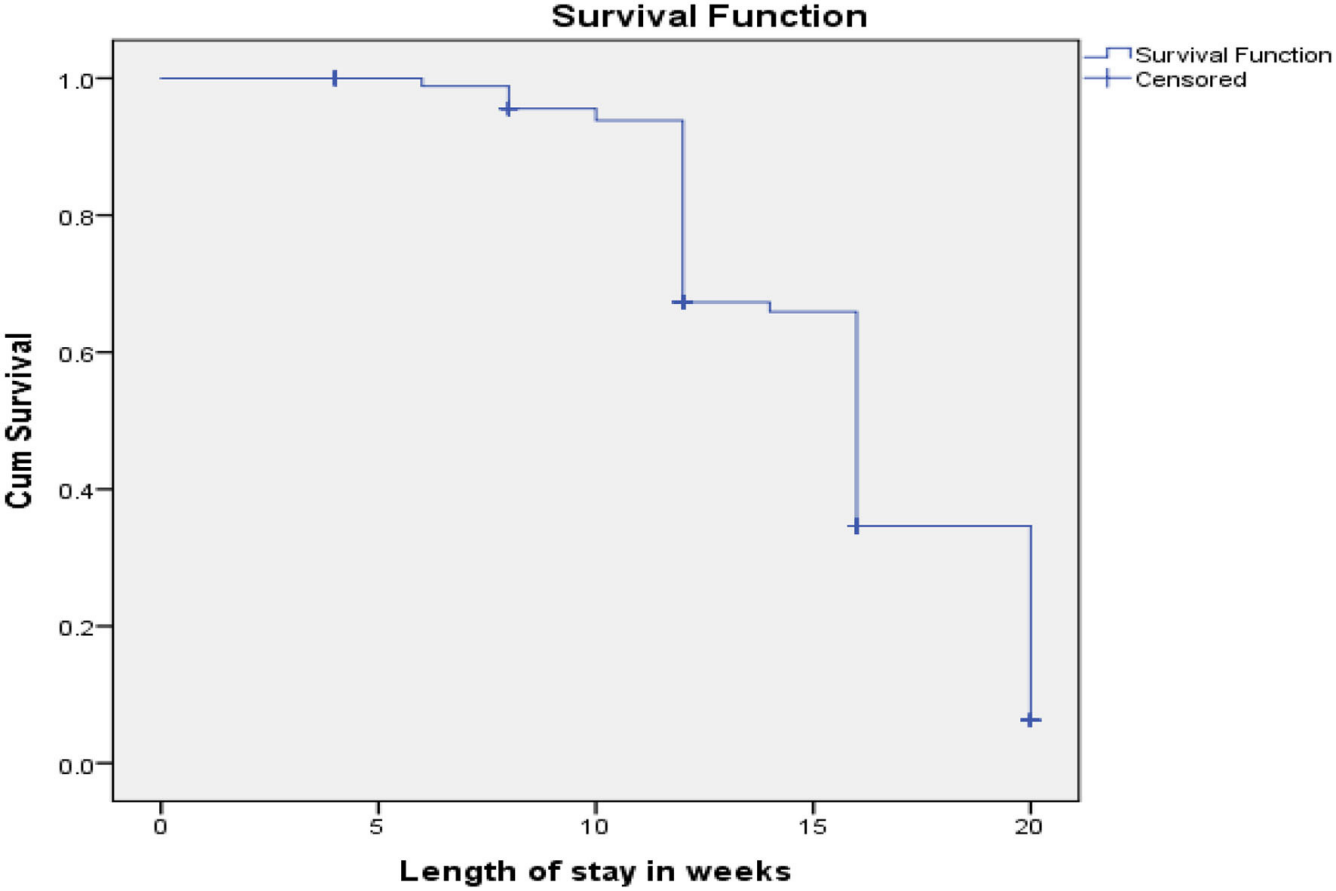
Kaplan Meier recovery estimate of children 6–59 months of age enrolled in targeted supplementary feeding program in Darolebu District, Oromia Region, Eastern Ethiopia, 2020.

A total of three hundred ninety-four (73%) (95% CI 69.4–76.4%), children recovered from moderate acute malnutrition. Above a quarter (27%) were censored (24.8% defaulters and 2.2% non-responders). Out of those who recovered, 319 (81%), had recovered within 16 weeks, while the rest (19%) recovered after 16 weeks. According to the Kaplan–Meier survival estimation, the median recovery time was 16 weeks (95% CI 15.4–16.6) (Figure 1). The recovery rate for children who used ready-to-use supplementary food (RUSF) was 82.8% whereas the recovery rate for those who used corn-soy blend with added micronutrients (CSB++) was 45.8%.

### Predictors of Time to Recovery Among the Study Participants

Initially, the socio-demographic, health services, and household characteristics of the study subjects were analyzed with a bivariate Cox regression model to determine predictors of time to recovery from moderate acute malnutrition. Accordingly, educational status of primary caregiver, number of household members, admission MUAC, vitamin-A supplements, deworming, admission status, follow-up status, distance to the treatment center, transportation access to the treatment center, measles vaccination, exclusive breastfeeding, complementary feeding, type of treatment foods used, sharing treatment food with family members, latrine availability, source of water, diarrhea, and food-security status were identified as predictors of time to recovery from moderate acute malnutrition. Then variables with a *P*-value <0.05 in bivariate Cox regression were fitted into a multivariable Cox regression model. Finally, in multivariable Cox-regression analysis, being in the age range of 24–59 months, having had mid-upper arm circumference (MUAC) at admission between 11.5 and 11.9 cm, had not dewormed, walking for more than an hour to receive the services, using ready-to-use supplementary food, and being from food-insecure family were significantly associated with time to recovery from moderate acute malnutrition at a value of *P* <0.05 (Table 5).

**Table 5:** Bivariate analysis of predictors of time to recovery from moderate acute malnutrition among children 6–59 months of age enrolled in targeted supplementary feeding program in Darolebu district, Eastern Ethiopia, 2020 (*N* = 540).

**Variables**	**Follow up outcomes**		**Crude HR (95%CI)**	***P*-value**
	**Recovered *N* (%)**	**Censored *N* (%)**		
**Age (in months)**				
6–23	158(56)	124(44)	1	
24–29	236(91.5)	22(8.5)	1.36(1.11–1.66)	0.042^*^
**Sex of the child**
Male	207 (73.9)	73 (26.1)	1	
Female	187 (71.9)	73 (28.1)	0.85(0.69–10.03)	0.095
**Residence**				
Urban	91 (67.4)	44(32.6)	1	
Rural	303 (74.8)	102 (25.2)	1.22(0.96–10.55)	0.096
**Educational status of caregiver**				
No formal education	145(51.6)	136(48.4)	1	
Primary school	162(96.4)	6(3.6)	1.50(1.19–10.88)	0.001^*^
Secondary school and above	87(95.6)	4(4.4)	1.31(1.00–1.70)	0.050^*^
**Occupation of caregiver**				
Housewife	353(73.8)	125(26.2)	1	
Civil servant	7(77.8)	2(22.2)	0.92(0.43–10.94)	0.788
Merchant	34(64.2)	19(35.8)	0.89(0.62–1.26)	0.510
**Responsible person with primary child care**				
Mother	314(73.5)	113(26.5)	1	
Father	80(70.8)	33(29.2)	1.08(0.84–1.38)	0.550
**Household member**				
1–5 members	134(80.7)	32(19.3)	1.25(1.01–1.54)	
≥6 members	260(69.5)	114(30.5)	1	0.039^*^
**Admission in MUAC**				
11–11.4 cm	156(57.8)	144(42.2)	1	
≥11.5 cm	238(88.1)	32(11.9)	1.37(1.12–1.68)	0.002^*^
**Vitamin A supplement**				
Yes	292(76.8)	88(23.2)	1	
No	102(63.8)	58(36.2)	0.77(0.61–0.96)	0.022^*^
**Received Deworming**				
Yes	276(80.5)	67(19.5)	2.03(1.46–2.81)	
No	118(59.9)	79(40.1)	1	0.001^*^
**Admission status**				
New admission	354(73.1)	130(26.9)	1.39(1.01–1.94)	
Re-admission	40(71.4)	16(28.6)	1	0.065
**Follow-up status**				
Regular	358(94)	23(6)	1	
Irregular	36(22.6)	123(77.4)	0.22(0.16–0.31)	0.001^*^
**Time taken to receive services from the nearby health post**				
≤ 60 min	251(71.1	102(28.9)	1.45(1.01–2.06)	
>60 min	143(76.5)	44(23.5)	1	0.042^*^
**Caregiver received health education**				
Yes	239(65.5)	126(34.5)	1	
No	155(88.6)	20(11.4)	1.19(0.98–1.46)	0.085
**Caregiver demonstrated cooking**				
Yes	127(72.2)	49(27.8)	1	
No	267(73.4)	97(26.6)	0.94(0.76–1.16)	0.552
**Measles vaccination**				
Yes	249(72.2)	96(27.80)	1.24(1.01–1.53)	
No	145(74.4)	50(25.6)	1	0.125
**Exclusive breastfeeding**				
Yes	297(74.3)	103(25.8)	1	
No	97(69.3)	43(30.7)	0.78(0.64–0.98)	0.238
**Complementary feeding**				
Yes	305(73)	113(27)	1.31(1.03–1.66)	
No	89(73)	33(27)	1	0.633
**Specialized nutritious foods used**				
CSB++	66(45.8)	78(54.2)	1	
RUSF	328(82.8)	68(17.2)	1.42(1.09–1.85)	0.010^*^
**Ration used only for MAM children**				
Yes	253(74.4)	87(25.6)	1.26(1.03–1.55)	
No	141(70.5)	59(29.5)	1	0.027^*^
**Diarrhea**				
Yes	111(51.9)	103(48.1)	1	
No	283(86.8)	43(13.2)	1.29(1.04–1.61)	0.023^*^
**Cough**				
Yes	63(72.4)	24(27.6)	1	
No	331(73.1)	122(26.9)	0.98(0.75–1.28)	0.854
**Food security status**				
Food secured	163(96.4)	6(3.6)	2.03(1.57–2.64)	
Food-insecure	231(62.3)	140(37.7)	1	0.001^*^

* *Significant at P-Value < 0.05*.

Children who are between the age range of 24 and 9 months were 1.24 times (AHR = 1.24, 95% CI: 1.01–1.54) more likely to recover faster than those between 6 and 23 months. Children who had admission MUAC between 11.5 and 11.9 cm were 1.27 times (AHR = 1.27, 95% CI: 1.03–1.56) more likely to recover faster than those who had admission MUAC between 11 and 11.4cm. The study subjects who were dewormed were 1.87 times (AHR = 1.87, 95%CI: 1.34–2.61) more likely to recover earlier than their counterparts. Similarly, children whose primary caregivers walked for an hour or less to receive the services were 1.2 times (AHR = 1.2, 95%CI: 1.02–1.99) more likely to recover faster compared to those whose primary caregivers walked for more than an hour to receive the services. Children who had received RUSF were 1.32 times (AHR = 1.32, 95%CI: 1.01–1.73) more likely to recover faster than those who had received CSB++. Children from food-secured families were 1.8 times (AHR = 1.8, 95%CI: 1.38–2.39) more likely to recover faster compared to their counterparts (Table 6).

**Table 6:** Predictors of time to recovery from moderate acute malnutrition among children 6–59 months of age enrolled in targeted supplementary feeding program in Darolebu District, Oromia Region, Eastern Ethiopia, 2020 (*N* = 540).

**Variables**	**Follow up outcomes**		**CHR (95% CI)**	**AHR (95% CI)**	***P*-value**
	**Recovered *N* (%)**	**Censored** ***N* (%)**			
**Age (in months)**					
6–23	158(56)	124(44)	1	1	
24–29	236(91.5)	22(8.5)	1.36(1.11–1.66)	1.24(1.01–1.54)	0.042^*^
**Admission in MUAC**					
11–11.4 cm	156(57.8)	144(42.2)	1	1	
≥11.5 cm	238(88.1)	32(11.9)	1.37(1.12–1.68)	1.27(1.03–1.56)	0.024^*^
**Vitamin A supplement**					
Yes	292(76.8)	88(23.2)	1	1	
No	102(63.8)	58(36.2)	0.77(0.61–0.96)	0.89(0.70–1.13)	0.348
**Received deworming**					
Yes	276(80.5)	67(19.5)	2.03(1.46–2.81)	1.87(1.34–2.61)	
No	118(59.9)	79(40.1)	1	1	0.001^*^
**Admission status**					
New admission	354(73.1)	130(26.9)	1.39(1.01–1.94)	1.37(0.98–1.92)	
Re-admission	40(71.4)	16(28.6)	1	1	0.065
**Time taken to receive services from the nearby health post**					
≤ 60 min	251(71.1)	102(28.9)	1.45(1.01–2.06)	1.2(1.02–1.99)	
>60 min	143(76.5)	44(23.5)	1	1	0.042^*^
**Measles vaccination**					
Yes	249(72.2)	96(27.80)	1.24(1.01–1.53)	1.18(0.96–1.45)	
No	145(74.4)	50(25.6)	1	1	0.125
**Exclusive breastfeeding**					
Yes	297(74.3)	103(25.8)	1	1	
No	97(69.3)	43(30.7)	0.78(0.64–0.98)	0.84(0.63–1.12)	0.238
**Complementary feeding**					
Yes	305(73)	113(27)	1.31(1.03–1.66)	1.08(0.79–1.47)	
No	89(73)	33(27)	1	1	0.633
**Specialized nutritious foods used**					
CSB++	66(45.8)	78(54.2)	1	1	
RUSF	328(82.8)	68(17.2)	1.42(1.09–1.85)	1.32(1.01–1.73)	0.044^*^
**Food security status**					
Food secured	163(96.4)	6(3.6)	2.03(1.57–2.64)	1.8(1.38–2.39)	
Food-insecure	231(62.3)	140(37.7)	1	1	0.001^*^

* *Significant at P-Value < 0.05 for AHR; CI, Confidence Interval; CHR, Crude Hazard Ratio; AHR, Adjusted Hazard Ratio; CSB++, Corn-soy blend with added micronutrients, RUSF, Ready-to-use supplementary food*.

## Discussion

This study aimed to determine treatment outcomes and predictors of time to recovery from MAM among children 6–59 months of age in Darolebu district, eastern Ethiopia. The study found a recovery rate of 73% (95% CI 69.4–76.4%) with 16 weeks as the median recovery. The finding implies that nearly a quarter of children in the study area are at risk of mortality and morbidity associated with moderate acute malnutrition. This result urges the government and stakeholders to devise tailored interventions to reduce the burden of acute malnutrition through early screening, accessing services closer to the community, supporting food-insecure households, monitoring sharing of treatment food at the household level after distribution, and timely provision of routine medication to all children to reach the national and global target of child mortality reduction attributed to malnutrition (19, 33).

The current study found that the overall recovery rate from MAM among the study participants was 73%, which was slightly below the minimum acceptable threshold for the international standard of 75% (1). The recovery rate in the current study is also consistent with the findings of the studies done in Southern Ethiopia and rural Burkina-Faso, which reported 73% and 74.5% recovery rates (34, 35), respectively. However, it is lower than the findings of the studies conducted elsewhere, for instance, 82.8% in West Arsi, Ethiopia (30), 77.5% in Rwanda (31), and 95.5 in Niger (36). This variation might be due to differences in guidance and support for TSFP among the study settings, and adherence to MAM management protocol across the regions. Different programming approaches have been found to affect the treatment outcome of MAM (13). For example, the management approach for MAM in Niger offered curative services for HIV, malaria, and some endemic illnesses (36), which could be claimed to have a higher recovery rate compared to the current study.

In the current study, the age of the child was identified as a predictor of time to recovery. Children between the age range of 24 and 59 months were 1.24 times (AHR = 1.24, 95% CI: 1.01–1.54) more likely to recover faster than those between 6 and 23 months. The finding is relatively supported by the results of the study conducted in Jimma, which reported that the recovery time of children <24 months was delayed by 22% (AHR = 0.78, 95% CI: 0.64–0.96) compared to their counterparts (10). This might be due to the mother being focused only on breastfeeding and may be reluctant to provide supplementary foods, especially if cooking nutritious foods (CSB++).

Deworming was identified as a significant predictor of time to recovery in the current study. Children who were not dewormed were 1.87 times (AHR = 1.87, 95%CI: 1.34–2.61) more likely to recover faster than their counterparts. The finding has supported the study conducted in Dire Dawa, Ethiopia, and Rwanda, which reported that children who had taken deworming were more likely to recover faster compared to those who had not taken it (23, 31). This might be because children with malnutrition are at an increased risk of infection and intestinal inflammation (37), which impair nutrient absorption and could delay the recovery time among children who had not taken deworming medication.

As found in this study, MUAC at admission was another predictor of time to recovery. Children who had admission MUAC between 11.5 and 11.9 cm were 1.27 times (AHR = 1.27, 95% CI: 1.03–1.56) more likely to recover faster than those who had admission MUAC between 11 and 11.4 cm. This is in line with the studies conducted in West Arsi, and Jima, Ethiopia, which reported that children who had good MUAC at admission had more likely recovered faster than their counterparts (10, 30). Mid-upper arm circumference measurement indicates the status of malnutrition and children who had good MUAC at admission may have a greater chance of good treatment outcome and the likelihood of early recovery than children with deteriorated MUAC at admission. This underpins the importance of early screening for moderate acute malnutrition and enrolling in the program to get a better outcome, faster recovery, and minimize short and long-term consequences of MAM.

This study also revealed that the time taken to receive the TSFP services from the nearby health post was significantly associated with the time to recovery. Children whose primary caregiver walked for an hour or less to receive the services were 1.2 times (AHR = 1.2, 95% CI: 1.02–1.99) more likely to recover faster compared to those whose primary caregiver walked for more than an hour to receive the services. This is supported by the study conducted in Shebedino, Southern Ethiopia, which showed that children whose families had walked for more than an hour to receive the services were 31% less likely to recover earlier than children whose families had walked for an hour or less to receive the services from the nearby health post (25). A possible explanation for this could be that caregivers who have limited access to the targeted supplementary feeding program site may delay in deciding to bring their child to the treatment center, and this may compromise the treatment success, including faster recovery. This implies that making the targeted supplementary feeding program services accessible to the community may help to improve adherence to the services and treatment outcome of MAM.

The current study revealed that the type of treatment foods received was another predictor of recovery time. Children who had received RUSF were 1.32 times (AHR = 1.32, 95%CI: 1.01–1.73) more likely to recover faster than those who had received CSB++. This finding is consistent with the results of studies conducted in Cameroon (38), Malawi (39), Southern Ethiopia (34), and West Arsi, Ethiopia (30), which reported children who were treated with RUSF had recovered earlier from MAM compared to those who were treated with CSB++. The possible explanation for the variation seen between the two treatment foods might be attributed to the dissimilarity nature between foods. Ready-to-use supplementary food (RUSF) requires no cooking, while CSB++ requires cooking and thus may be shared with other members of a household. Thus, a child whose primary caregiver is busy and/or unskilled in cooking as well as sharing CSB++ with other members of the household could not get the adequate amount of energy required on a daily basis, which could further claim for delayed recovery among children treated with CSB++.

Moreover, this study revealed that household food insecurity was significantly associated with recovery time. Children from a household with food secured were 1.8 times (AHR= 1.8, 95%CI: 1.38–2.39) more likely to recover earlier compared to their counterparts. This finding is supported by the study conducted in West Arsi, and Shebedino, Ethiopia, which reported that recovery time had been delayed by 51% and 53% for children from severe food-insecure families, respectively (25, 30). This may be due to household food insecurity, which may prompt mothers to share specialized nutritious food with other members of the household and sell some parts of foods used for other purposes in the house.

## Conclusion

The study found the recovery rate was slightly below the accepted minimum international standard. Age of the study subjects, MUAC at admission, deworming status, time taken to receive the services from the nearby health post, varieties of nutritious foods used for treatment, and household food security status were found to be significant predictors of recovery time from moderate acute malnutrition. In general, further work is needed to improve the treatment outcomes and reduce mortality and morbidity attributed to moderate acute malnutrition. Furthermore, prospective studies are required to incorporate parameters that are not addressed in the current study.

### Strength and Limitations of the Study

The study explored parental and child conditions through direct interviews rather than solely relying on record review. Despite this, we acknowledge a few limitations in our study. First, we failed to consider the seasonality pattern of acute malnutrition, which may have introduced a slight selection bias to the study. Second, this study might be exposed to recall bias since some questions were based on the caregiver's memory. Third, this study recruited a limited sample size, and hence the conclusions of this study may not directly represent children enrolled in TSFP in the other areas of Ethiopia.

## Data Availability Statement

The raw data supporting the conclusions of this article will be made available by the authors, without undue reservation.

## Ethics Statement

The study was approved by the Institutional Health Research Ethics Review Committee, College of Health and Medical Sciences, Haramaya University, Ethiopia with approval number (Ref. no. IHRERC/162/2020). Written and verbal informed consent to participate in this study was provided by the participants' legal guardian/next of kin.

## Author Contributions

MR, JK, LO, and MD made significant contributions to the conception of the idea and design, participated in proposal development and data collection, and analyzed and interpreted the data. JK wrote the original draft of the manuscript. JK, LO, and MD reviewed and edited the manuscript for important intellectual content. All authors read and approved the final manuscript.

## Conflict of Interest

The authors declare that the research was conducted in the absence of any commercial or financial relationships that could be construed as a potential conflict of interest.

## Publisher's Note

All claims expressed in this article are solely those of the authors and do not necessarily represent those of their affiliated organizations, or those of the publisher, the editors and the reviewers. Any product that may be evaluated in this article, or claim that may be made by its manufacturer, is not guaranteed or endorsed by the publisher.

## Abbreviations

AHR: Adjusted Hazard Ratio
CI: Confidence Interval
CHR: Crude Hazard Ratio
CSA: Central Statistical Agency
CSB++: Corn-Soy Blend with added micronutrients
RUSF: Ready-to-Use Supplementary Foods
MAM: Moderate Acute Malnutrition
SPSS: Statistical Package for Social Science.
